# A new method in the synthesis of conductive polymers; synthesis, characterization, and investigation of photocatalytic properties of polyaniline

**DOI:** 10.55730/1300-0527.3559

**Published:** 2023-03-31

**Authors:** Muzaffer CAN

**Affiliations:** Department of Chemistry, Kırıkkale University, Kırıkkale, Turkey

**Keywords:** Conducting polymers, polyaniline, zinc ion, PSS, Lewis acid

## Abstract

As known, in the synthesis of conductive polyaniline polymer from aniline monomer, the polymerization medium must be acidic. In order to make the polymerization medium acidic, protonic acids such as HCl and H_2_SO_4_ are generally used. In this study, two acids have been used to make the polymerization medium acidic. One of them, as we know, is H_2_SO_4_ as a protonic acid. The other acid is the zinc ion, which is a Lewis acid. The metal ion has never been used for this purpose up to now. Two polyaniline polymers have been synthesized to prove that conductive polymers can also be synthesized in the presence of metal ions instead of protonic acid. One of them is the polyaniline polymer, synthesized in the presence of H_2_SO_4_(PANI). The other polymer is polyaniline polymer, synthesized in the presence of zinc ions as acid (PANI-Zn-PSS). The synthesized polymers have been characterized by using scanning electron microscopy (SEM), energy dispersive X-ray spectrometry (EDX), X-ray diffraction diffractometry (XRD), Fourier transform infrared spectroscopy (FTIR), Ultraviolet-visible spectrophotometer (UV-vis.), thermal analysis techniques (thermo-gravimetric analysis, TGA)/differential thermal analysis, DTA) and electrical conductivity measurements kit. The photocatalytic activities of polymers synthesized have been investigated by the degradation of methylene blue (MB) dye in aqueous medium under UV light.

## 1. Introduction

Ease of processing, mechanical behavior, flexibility, and low density are important properties of polymers. Conducting polymers have a special place and importance among polymers.

Conducting polymers are similar to metals in their electronic conductivity properties. For this reason, they are called synthetic metals. The most widely studied conductive polymers are polyaniline, polypyrrole, polythiophene, and their derivatives. These conductive polymers were synthesized using aniline, pyrrole, and thiophene monomers [1–5]. Among these conductive polymers, polyaniline is the most widely studied one because of its environmental stability, high and controlled conductivity values, electrical and optical properties, unique redox behavior, and ease of preparation. PANI is used in the fields such as electrochromic devices, light-emitting diodes, rechargeable batteries, electronic circuits, membranes and anticorrosion paints [6–15]. In some cases, purehomopolymers alone may not be sufficient for use as polymer materials. In such cases, the synthesis of new polymers, copolymers, or composites can become a new research topic. Synthesizing polymers in new and different environments apart from known methods may be a new research topic as in this study.

Polyaniline has specific reversible protonation-deprotonation (doping and dedoping) properties. In other words, emeraldine base (EB) and emeraldine salts (ES, polaronic and/or bipolaronic) can be converted to each other by means of an acid and a base [16, 17]. In this study, aniline and polyaniline were chosen as a model compound because it could be protonated more easily than other polymers and monomers (metal addition to them would also become easy). Some studies on PANI-metal doping had been carried out by our study group [18–21].

In the synthesis of polyaniline polymer from aniline and its derivatives, the polymerization medium must be acidic. Many protonic acids such as HCl and H_2_SO_4_ have been used to acidify the polymerization medium. Whether metal ions with Lewis acid properties could be used instead of protonic acid to acidify polymerization environments was investigated in this study. As it is known, changing the polymerization medium changes the chemical structure of the synthesized polymers and, accordingly, their properties. For this reason, polyaniline polymers were synthesized under two different conditions. One of the conditions was media consisting of aniline monomer, H_2_SO_4_, sodium polystyrene sulfonate (PSS) and oxidant (PANI). The other was the medium consisting of aniline monomer, Zn^2+^, PSS, and oxidant (PANI-Zn-PSS). The synthesized polymers were characterized by SEM, XRD, EDX, FTIR, UV-vis, TGA, DTA, and electrical conductivity measurements. In addition, the photocatalytic activities of the polymers we synthesized were also investigated.

## 2. Materials and methods

### 2.1. Materials

The aniline (98%, Aldrich) used was distilled under vacuum before use and its solution in acetonitrile was prepared and stored in the dark at 5 °C. Acetonitrile (99.99%, Sigma-Aldrich) was used as solvent. H_2_SO_4_ (98%, Aldrich), a strong acid in aqueous and acetonitrile media, was used to acidify the polymerization medium. Ammonium peroxidisulfate, (NH_4_)_2_S_2_O_8_ (99.5%, Aldrich) was used as an oxidant. Zinc nitrate, Zn(NO_3_)_2_ (99%, Aldrich), was used as the source of Zn^2+^. Sodium polystyrene sulfonate (PSS) was purchased from Aldrich. Methylene blue (≥ 95%) was purchased from Sigma-Aldrich. Except for aniline, all chemicals used in the experiment were analytical grade and used without any processing as taken.

### 2.2. Characterization

The UV–vis spectra of the polymers were performed in the range of 200–800 nm using a Perkin Elmer Lambda-35 UV–vis. spectrophotometer. FT-IR spectra of the polymers were recorded in the range of 2000–400 cm^−1^ using a Jasco FTIR-430 Fourier transform infrared spectrometer. X-ray diffraction device, Rigaku D/MAX-2200 device, was used to determine the phases and diffraction peaks of PANI and PANI-Zn-PSS polymers. The analysis was carried out with CuKα wavelength radiation beam at room temperature with 10° ≤ 2θ ≤ 80° limit values. The thermal analysis curves (TGA and DTG) were obtained using a PerkinElmer diamond thermal analyzer with a sample size of 5–10 mg and a heating rate of 10 °C min^−1^ under nitrogen atmosphere. The temperature scanning speed was 10 °C/min. For SEM/EDX measurements, polymer samples were subjected to a thin gold coating by using a FEI brand, Quanta 400 FEG model SEM fine coater. The dry conductivity values of polymers were measured by using a four-probe electrical conductivity measuring device, Entek Electronic, at room temperature. The photocatalytic activities of polymers were measured by using the UV-C tube lamp in a quartz tube (properties of the lamp: 15 W, 41 cm length, 2.5 cm radius Philips Netherlands G15T8 model unfiltered lamp).

### 2.3. Synthesis of PANI polymer

**Scheme 1:**
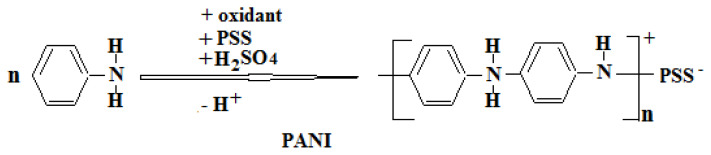


PANI was synthesized by chemical polymerization of aniline monomer using (NH_4_)_2_S_2_O_8_ oxidant. The amounts of monomer, acid, and oxidant used in the synthesis are chemically equal to each other. Two different solutions were prepared. One of these solutions contains aniline, PSS and H_2_SO_4_. The other solution contains only the oxidizing agent. Afterwards, the solution containing the oxidant was added dropwise on the other solution and, mixed with a magnetic stirrer until solution homogeneity was achieved. After 24 h, the formed polymer was filtered, washed and dried. The mechanism proposed for the synthesis of PANI polymer is given below (Scheme 1).

### 2.4. Synthesis of PANI-Zn-PSS polymer

**Scheme 2:**
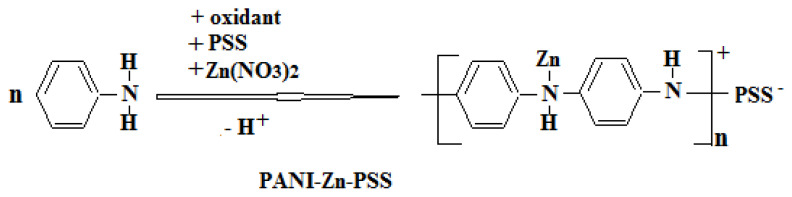


Firstly, a solution containing aniline, Zn(NO_3_)_2_, and PSS was prepared. Next, the solution containing oxidant was added dropwise to this solution. The final solution was kept in the dark for 24 h to complete the polymerization reaction. After 24 h, the polymer was filtered, washed, and dried. The mechanism proposed for the synthesis of PANI-Zn-PSS polymer is given below (Scheme 2).

### 2.5. Measurements of photocatalytic activity

In each experiment, 3.2 mg of a catalyst was added into the mixture containing 6 mL of MB dye and the absorbance of each mixture was measured at certain times. After each measurement, the catalyst was separated from the dye by centrifugation and washed several times with pure water and dried at 100 °C. The photocatalytic degradation of the MB dye was studied at 25 °C. The degradation percent of MB cell was determined by measuring the absorption at its characteristic wavelength via UV-vis spectroscopy. The degradation percentage of the MB dye was calculated using the following equation:


Percent of degradation (%)=[(Co-C)/Co]×100,

where Co and C represent the concentration of the dye before irradiation and after irradiation time, respectively.

## 3. Results and discussion

### 3.1. UV-vis spectroscopy studies

**Figure 1:**
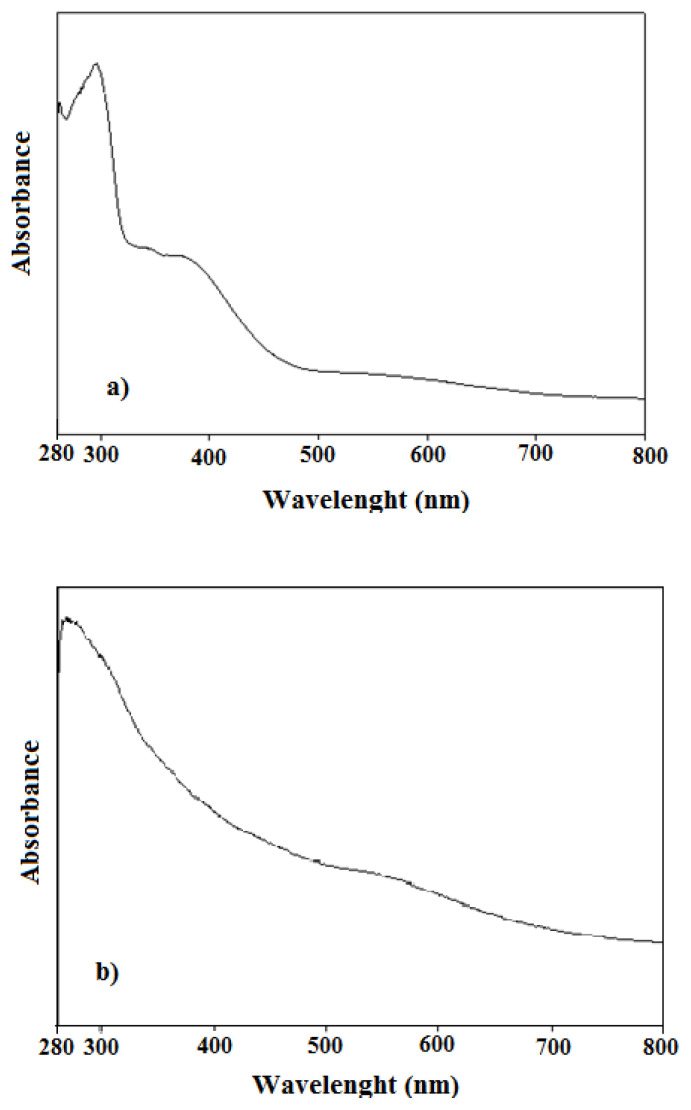
UV-vis spectra of a. PANI; b. PANI-Zn-PSS polymers.

Synthesized polymers were dissolved in N-methyl-2-pyrrolidone. UV-vis spectra of each polymer solution were then taken (Figures 1a and 1b). There are two absorption bands in the spectrum of the PANI polymer at 295 nm and 375 nm (Figure 1a). The band observed at 295 nm belongs to the π → π* transition of PANI polymer. It is suggested that the polyaniline polymer has absorption bands belonging to the π → π* transition around 290 nm [22–27]. It has also been suggested that the bands observed at these wavelengths belong to π → π* transitions in the polyaniline polymers in benzenoid form [28]. There is an absorption band at 286 nm in the spectrum of pure aniline [29]. It is probable that this band belongs to π → π * transitions of phenyl ring because there are no other functional groups to absorb. The phenyl ring in pure aniline and the phenyl ring in the polymer chain are different from each other in terms of chemical environment (aniline is in a single ring structure while polyaniline is in multiring structure). Therefore, there are differences between the absorption wavelengths of both substances. The band observed around 375 nm belongs to polaron-bipolaron transition of PANI polymer. It is also suggested that the bands observed around this wavelength belong to the polaron-bipolaron and even polaron-π* transition [30].

There are also two bands in the spectrum of the PANI-Zn-PSS polymer at 290 nm and 535 nm (Figure 1b). The band observed at 290 nm belongs to the π → π* transition, as in PANI. The band at 535 nm belongs to the π → π* transition of the quinoid polyaniline. It is suggested that polyaniline bands in quinoid form are observed above 500 nm [25].

### 3.2. FTIR spectroscopy studies

**Figure 2:**
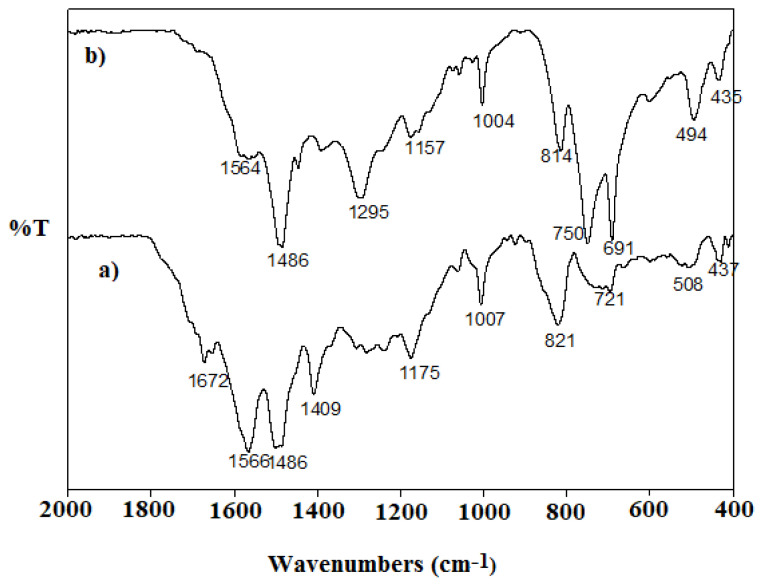
FT-IR spectra of a. PANI; b. PANI-Zn-PSS polymers.

FTIR spectra of the polymers synthesized were taken (Figures 2a and 2b) and the characteristic wave numbers belonging to the bands observed in the FTIR spectrum of each polymer are given in Table. The number of bands in both spectra is equal to each other. However, the wave numbers of the bands are different. The reason of the difference between the wave numbers is that the polymerization environments and therefore the chemical structures of the synthesized polymers are different from each other (PANI polymer has N-H group, PANI-Zn-PSS polymer has N-Zn group).

As is known, polyaniline polymer has two structural forms, which are quinoid and benzenoid. It is suggested that polyaniline polymers in quinoid form generally have an absorption band above 1566 cm^−1^, and the N=Q=N group, which is one of the most important groups of the quinoid structure, has an absorption band around 1120 cm^−1^ [31, 32]. In these studies, it has also been suggested that polyaniline polymers in benzenoid form have absorption bands in the range of 1480–1566 cm^−1^ (Figure 2a). There are 1566 cm^−1^ and 1486 cm^−1^ absorption bands in the FTIR spectrum of the synthesized polymer. There is also a band thought to belong to the C-N stretch at 1409 cm^−1^ (the wavenumber of this band has been suggested as 1378 cm^−1^ in the literature). The bands observed show that there are both benzenoid and quinoid units in the PANI structure. The 1007 cm^−1^ and the 1175 cm^−1^ bands belong to the aromatic C-H in-plane bending. Out-of-plane deformations of C-H are located in the region of 800–880 cm^−1^ [28]. In the literature, the bands observed in the wavenumbers less than 1200 cm^−1^ belong to in-plane and out-of-plane bending bands of C-C, N-H, C-H groups and also dopant materials [31–39].

There are no absorption bands above 1586 cm^−1^ and at 1409 cm^−1^ in the FTIR spectrum of the PANI-Zn-PSS polymer (Figure 2b). This indicates that quinoid units are almost absent in the PANI-Zn-PSS polymer and that this polymer is predominantly composed of benzenoid units. An important conclusion we can draw from FTIR studies is that the polyaniline polymer synthesized by using metal ions which is a Lewis acid instead of protonic acid is predominantly in benzenoid form.

### 3.3. XRD studies

**Figure 3:**
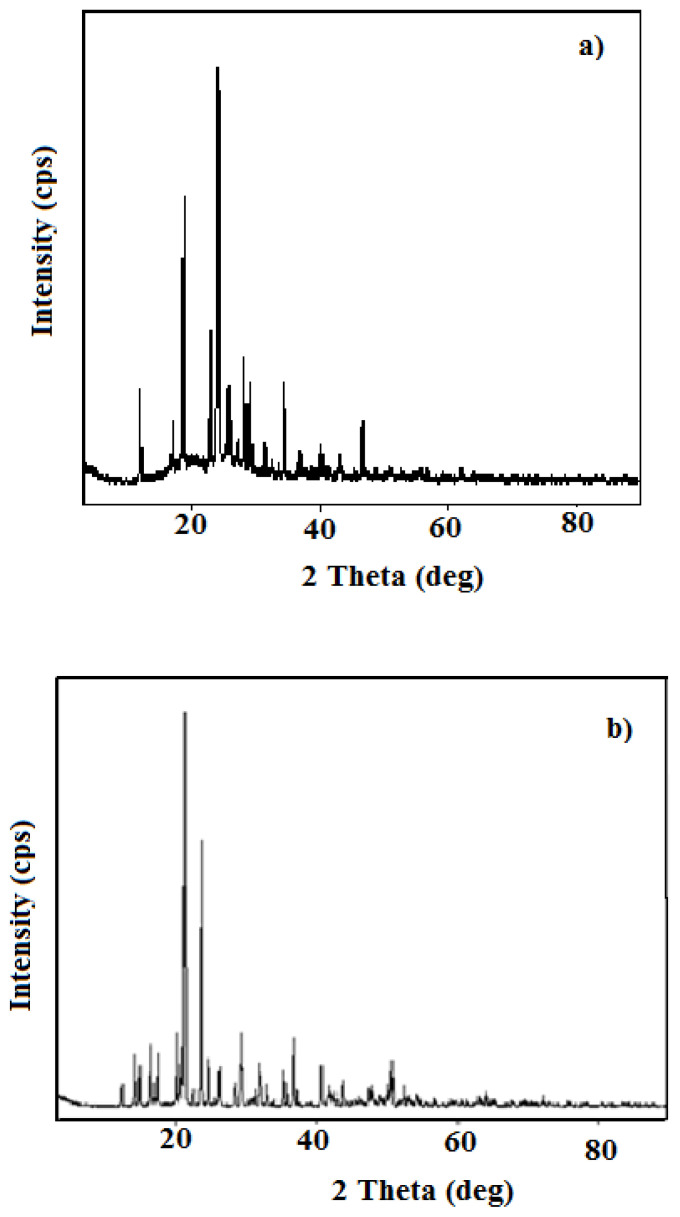
XRD diffraction patterns of a. PANI; b. PANI-Zn-PSS polymers.

In the present study, the crystallinity quality of the PANI polymer and the PANI-Zn-PSS composite and the crystallinity change according to the doped metal were investigated using powder XRD measurements. The diffraction patterns in the XRD diffractograms were recorded in the range of 3° to 90° and are shown in Figures 3a and 3b. The XRD diffraction pattern of polyaniline (Figure 3a) shows sharp diffraction peaks at around 2θ = 14°, 18°, 26°, 28°, 30°, 35°, and 46º belonging to the characteristic PANI diffraction peaks. The presence of characteristics XRD diffraction peaks shows that the degree of crystallinity is very high in this polymer. In the case of Zn-PSS polymer in the main PANI structure, there appear some shifts and new characteristic impurity peaks. This is attributed to the fact that the Zn-PSS structure is successfully inserted in the PANI crystal structure. The characteristic peaks in the XRD pattern of PANI-Zn-PSS are noted to occur at 12°, 18°, 24°, 26°, 28°, 30°, 34° and 44° (Figure 3b). It is clear that the presence of Zn in the polycrystalline system with a metal doping causes the appearance or disappearance of new diffraction peaks along with the changes in the intensity of the properties in XRD diffraction graphs. Also, the increase in the characteristic XRD peak numbers shows the growth of a different crystal system simultaneously in the PANI polymer. The number of peaks in both spectra is approximately equal, but the peaks differ from each other as shape. The reason for this difference is that the chemical structures of the polymers are different from each other. These results show that a polymer, which has similar crystalline properties to polyaniline synthesized in the presence of a protonic acid, can also be synthesized in the presence of a metal ion, which is Lewis acid.

### 3.4. Thermogravimetric studies

**Figure 4:**
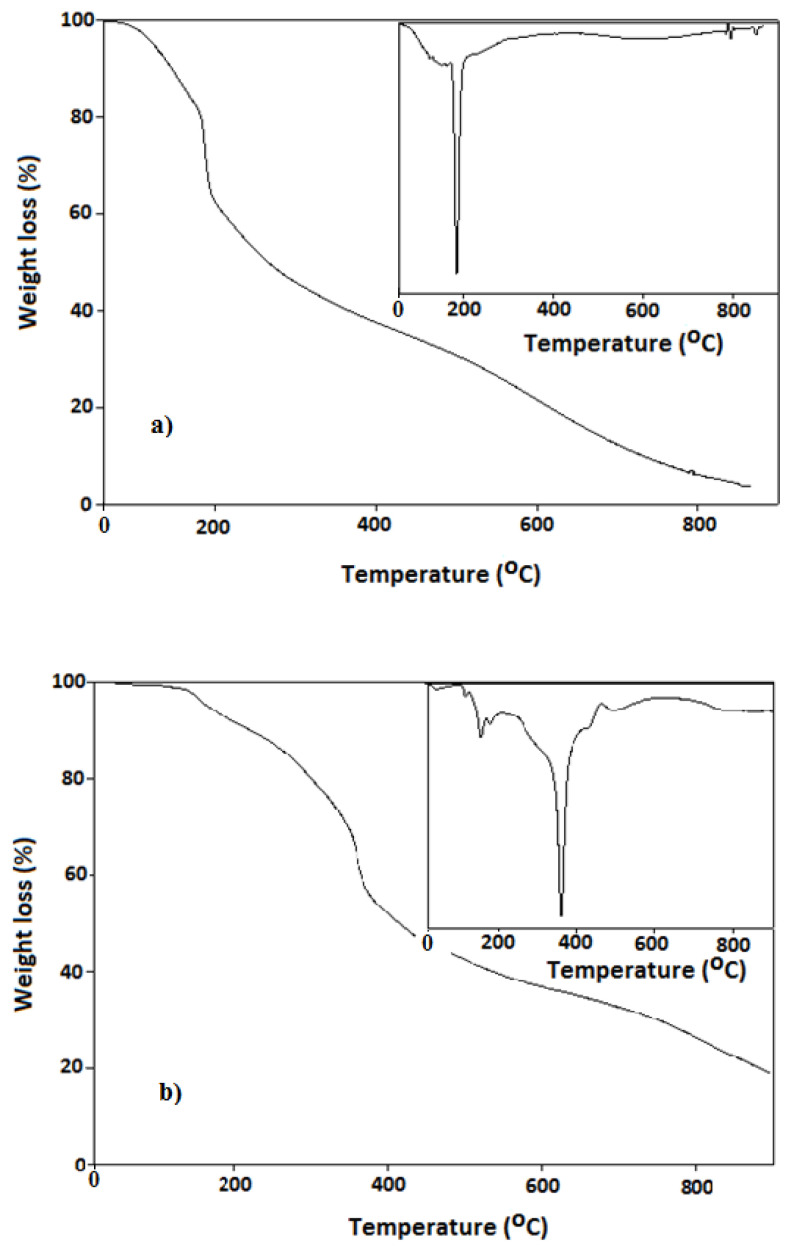
TGA and DTA curves of a. PANI; b. PANI-Zn-PSS polymers.

The thermal behavior of the synthesized polymers was also investigated. The thermal weight loss of the PANI polymer occurs around 190 °C (Figure 4a). The total weight loss observed at this temperature value is around 40% and this weight loss belongs to the degradation of the (+) charged units in the polymer chain [40]. The thermal weight loss of PANI-Zn-PSS polymer is observed around 380 °C (Figure 4b). This mass loss belongs to the degradation of (+) charged units in the PANI-Zn-PSS polymer, as in the PANI polymer. The total weight loss observed at this temperature value is around 60%. While the mass loss in PANI polymer is 40%, this is 60% in PANI-Zn-PSS. This case indicates that the (+) charged units in the PANI-Zn-PSS polymer are higher than the (+) charged units in the PANI polymer. (+) charged units in the PANI-Zn-PSS polymer are higher than that in the PANI polymer.

These thermal studies show that the thermal degradation temperatures of both polymers are different from each other. As highlighted in previous sections, this difference is due to the fact that polymers have different chemical structures. (PANI polymer has N-H unit, PANI-Zn-PSS polymer has N-Zn unit). An important result we will draw from thermal studies is that when the polymer is synthesized in the presence of Zn, this metal ion enters the polymer structure and increases the thermal stability of the polymer approximately two times. The reason for the thermal stability may be that the N-Zn bond is stronger than the N-H bond.

### 3.5. SEM and EDX studies

**Figure 5:**
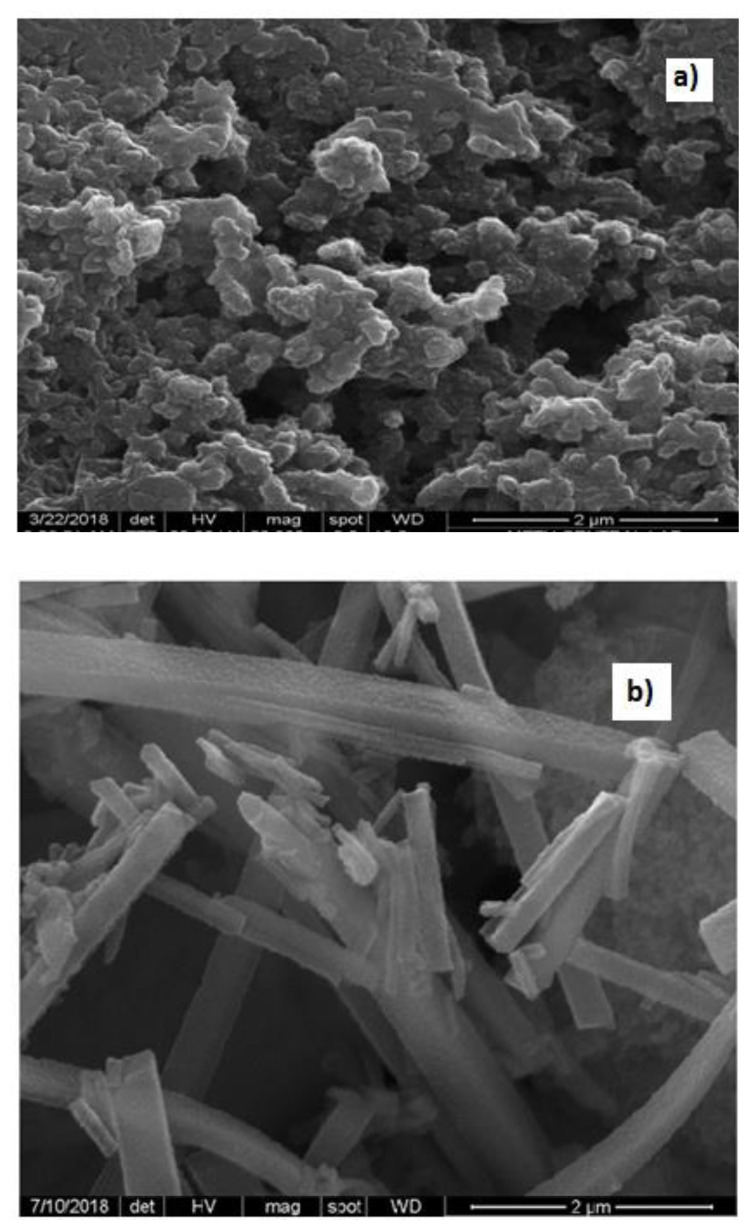
SEM patterns of a. PANI; b. PANI-Zn-PSS polymers.

SEM patterns were taken to explain the morphological structure of the synthesized polymer (Figures 5a and 5b). SEM patterns of the synthesized polymers are completely different from each other. In particular, SEM pattern (Figure 5b) of the polymer synthesized in the presence of Zn^2+^ ion show that the polymer synthesized in the presence of metal ions has a rod-like structure. Rod-like polymer has a more crystalline structure compared to the other polymers. XRD studies (Figure 3b) also showed that the polymer synthesized in the presence of Zn^2+^ ion had a better crystal structure. In both XRD and SEM analyses, crystallization behaviors supporting each other were obtained.

**Figure 6:**
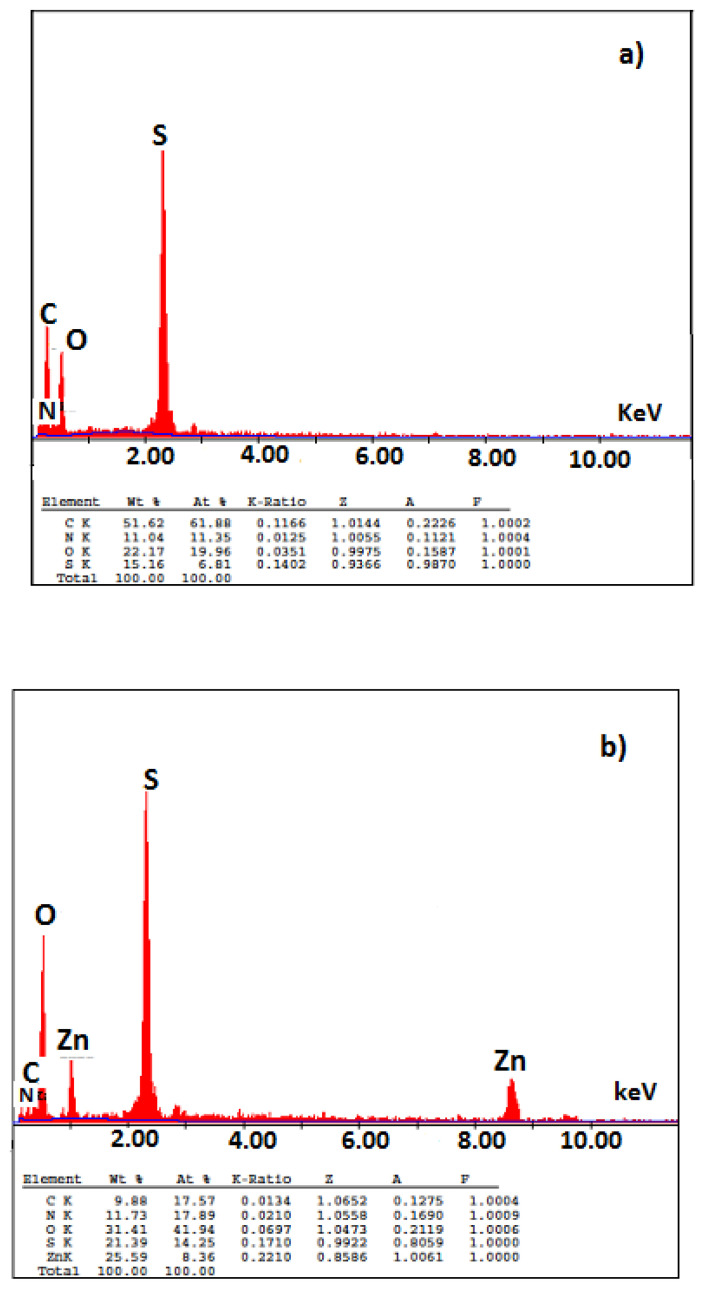
EDX spectra of a. PANI; b. PANI-Zn-PSS polymers.

EDX spectra of polymers were also taken to prove the presence of zinc in the PANI-Zn-PSS polymer structure and to determine the percentage of zinc and S in the polymer structure (Figures 6a and 6b). As can be seen from the figures, the amounts of S in the structure of PANI and PANI-Zn-PSS polymers are 15.16% and 21.39%, respectively, there is zinc in the PANI-Zn-PSS polymer structure and its amount is 25.59% (Figure 6b). An important result that we can obtain from these spectra is that the amount of sulfur in the synthesized polyaniline polymer increased when zinc ion was used. (S exists in the PSS structure used as dopant.) The increase in the amount of sulfur proportional to the amount of zinc indicates that the doping rate in the polyaniline polymer synthesized in the presence of Zn^2+^ increases (the amount of positive charge formed by the zinc ion on the polymer is greater than that of the proton).

### 3.6. Conductivity measurements

In DC conductivity measurements, dry pellets were prepared from powdery polymer material under a pressure of 5-ton cm^−2^. Gold-plate probes were used to avoid any errors caused by ohmic contacts. The resistivity of the samples was measured at five different positions and at least two pellets were measured for each sample. An average of 10 readings was done for conductivity calculations. The measured conductivity values for PANI and PANI-Zn-PSS polymers are 1.45 × 10^−1^ S/cm and 2.926 × 10^−4^ S/cm, respectively. The low conductivity in the PANI-Zn-PSS may be due to the (+) charged units (benzenoid structure) in the polymer structure being higher than the PANI polymer. Especially, UV studies support this view.

### 3.7. Photocatalytic degradation of methylene blue

Removal of dyes in wastewater is very important. Since these dyes are dissolved in wastewater, it is difficult to remove these dyes from these waters by mechanical means. However, these dyes can be removed with degradation of dyes. As we have seen from our previous studies, the degradation of dyes is 1% without using a catalyst [19, 41]. And again, we have seen from these studies that the rate of degradation increases when the catalyst is used. The catalyst (PANI and PANI-Zn-PSS) was added into 3 mL of the dye solution of MB and mixed. The solution was stirred with a magnetic stirrer before being placed under the light source and left in the dark for 15 min to reach an adsorption–desorption balance. It was then placed under UV light. The decomposition amount of MB in the absorption cell was determined by measuring the absorption at its characteristic wavelength via UV–vis spectrophotometer.

**Figure 7:**
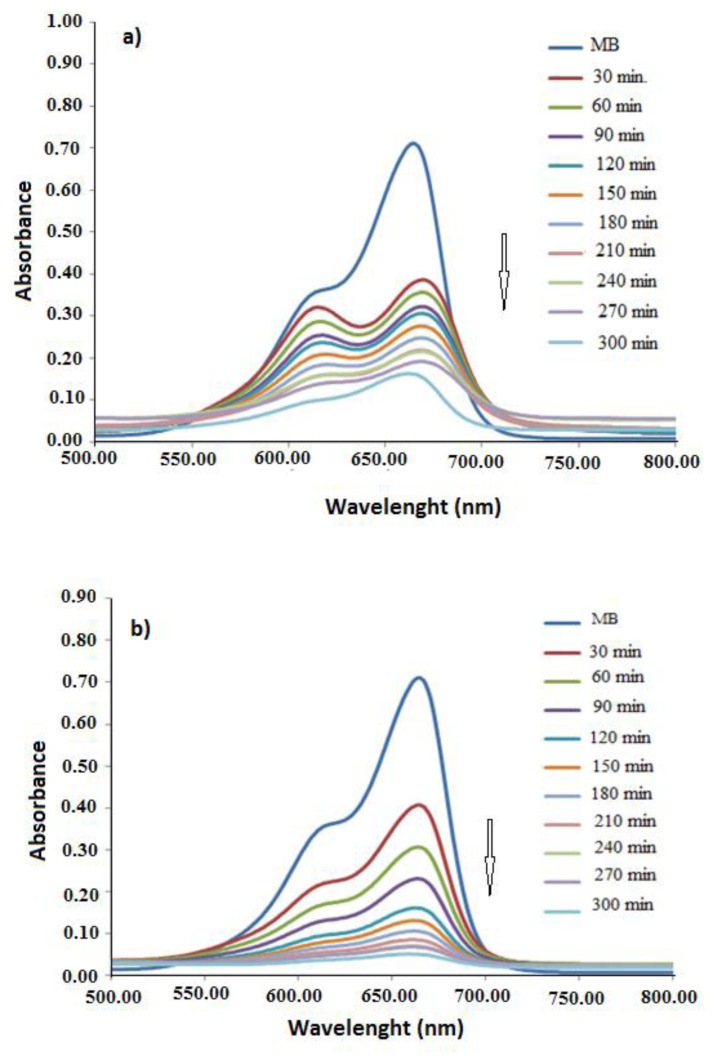
The absorption spectra taken at certain times of a. Pure MB and MB + PANI mixture; b. Pure MB and MB + PANI-Zn-PSS mixture.

The MB dye has two characteristic absorption bands at 640 nm and 664 nm (the first absorption bands that can be seen in each spectrum, given in Figures 7a and 7b, belong to pure MB). In order to investigate whether the polymers we synthesized show photocatalytic activity, the absorbances of the polymer-MB containing solutions were measured at different times. As seen in Figures 7a and 7b, the intensity of the absorption band of MB dye decreases in time when one of the polymers is present in the experimental medium. The decrease in the absorption band intensities of the dye indicated that dye has been degraded by PANI and PANI-Zn-PSS. After 300 min, while the intensity of the absorption bands of MB disappeared completely in the presence of PANI-Zn-PSS polymer, they partially decreased in the presence of the PANI polymer (300 min was determined by considering the time when MB is completely degraded in the presence of one of the polymers). This case shows that both polymers have photocatalytic activity and a photocatalytic property of PANI polymer is lower than that of the PANI-Zn-PSS polymer.

**Figure 8:**
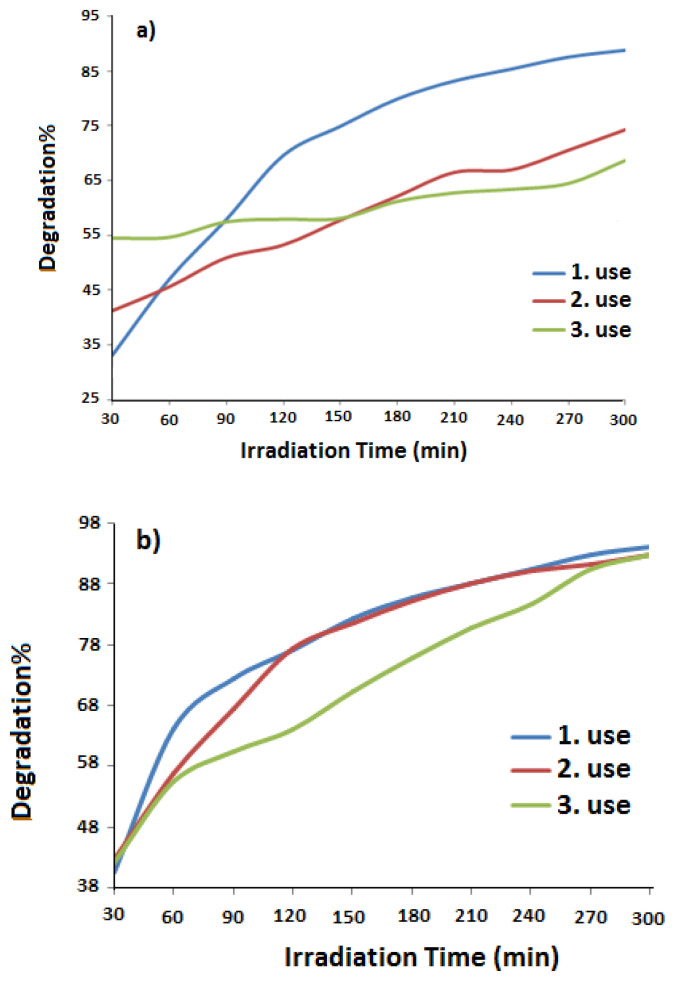
Reuses of a. PANI; b. PANI-Zn-PSS polymers as catalyst in photocatalytic degradation of MB.

Figures 8a and 8b show the reuse (three times) stabilities of PANI and PANI-Zn-PSS polymers under UV light. From these graphs, it is seen that the PANI-Zn-PSS polymer retains its photocatalytic activity after each use, while the PANI polymer loses it. These results show that the polymers synthesized in the presence of zinc ion have more stable photocatalytic activity.

## 4. Conclusion

In this study, PANI and PANI-Zn-PSS polymers were synthesized. All polymers synthesized were characterized by SEM, XRD, EDS, FTIR, UV-vis, TGA, DTA, and dry electrical conductivity measurements. Some important results have been obtained from experimental studies. We can list them as follows; i) conductive polyaniline polymers can also be synthesized in the presence of metal ions, which are Lewis acid properties instead of protonic acids, ii) the zinc ions can also participate in the polymer structure like protons, iii) when Zn^2+^ ion is used to make the polymerization medium acidic, the amount of zinc in the synthesized polymer structure is 25.59%, iv) synthesized polymers using metal ions instead of protonic acid have better crystal structures, v) PANI-Zn-PSS polymer has more stable photocatalytic properties than PANI polymer has.

## Figures and Tables

**Table:** Characteristic wave numbers of bands observed in FTIR spectra of polymers.

PANI (cm^−1^)	PANI-Zn-PSS (cm^−1^)
1672	**1564**
1566	**1486**
1486	**1295**
1409	**1157**
1175	**1004**
1007	**814**
821	**750**
721	**691**
508	**494**
437	**435**
